# A unified framework of immunological and epidemiological dynamics for the spread of viral infections in a simple network-based population

**DOI:** 10.1186/1742-4682-4-49

**Published:** 2007-12-20

**Authors:** David M Vickers, Nathaniel D Osgood

**Affiliations:** 1 Interdisciplinary Studies, College of Graduate Studies and Research, University of Saskatchewan, Saskatoon, Saskatchewan, Canada; 2 Department of Computer Science, College of Arts and Science, University of Saskatchewan, Saskatoon, Saskatchewan, Canada

## Abstract

**Background:**

The desire to better understand the immuno-biology of infectious diseases as a broader ecological system has motivated the explicit representation of epidemiological processes as a function of immune system dynamics. While several recent and innovative contributions have explored unified models across cellular and organismal domains, and appear well-suited to describing particular aspects of intracellular pathogen infections, these existing immuno-epidemiological models lack representation of certain cellular components and immunological processes needed to adequately characterize the dynamics of some important epidemiological contexts. Here, we complement existing models by presenting an alternate framework of anti-viral immune responses within individual hosts and infection spread across a simple network-based population.

**Results:**

Our compartmental formulation parsimoniously demonstrates a correlation between immune responsiveness, network connectivity, and the natural history of infection in a population. It suggests that an increased disparity between people's ability to respond to an infection, while maintaining an average immune responsiveness rate, may worsen the overall impact of an outbreak within a population. Additionally, varying an individual's network connectivity affects the rate with which the population-wide viral load accumulates, but has little impact on the asymptotic limit in which it approaches. Whilst the clearance of a pathogen in a population will lower viral loads in the short-term, the longer the time until re-infection, the more severe an outbreak is likely to be. Given the eventual likelihood of reinfection, the resulting long-run viral burden after elimination of an infection is negligible compared to the situation in which infection is persistent.

**Conclusion:**

Future infectious disease research would benefit by striving to not only continue to understand the properties of an invading microbe, or the body's response to infections, but how these properties, jointly, affect the propagation of an infection throughout a population. These initial results offer a refinement to current immuno-epidemiological modelling methodology, and reinforce how coupling principles of immunology with epidemiology can provide insight into a multi-scaled description of an ecological system. Overall, we anticipate these results to as a further step towards articulating an integrated, more refined epidemiological theory of the reciprocal influences between host-pathogen interactions, epidemiological mixing, and disease spread.

## Background

Epidemics consist of dynamic processes at multiple biological scales. From host-pathogen interactions to host-host interactions infectious diseases have had a major influence on the development of our immune systems and the evolution of human ecology [1,2]. In recent decades, remarkable advances in immunology and virology have provided fundamental insights into the detailed mechanisms of infection pathogenesis and immune recognition [3,4]. Meanwhile epidemiological modelling has enriched our understanding of the properties of infectious disease thus enabling humankind to better control its spread [2].

Within an individual host, a major factor governing infectious disease dynamics is how quickly and effectively the immune system can respond to infection (hereafter referred to as immune responsiveness) [1]. For clearing a viral infection, this is defined as the average rate at which naive CD8+ cells proliferate into cytotoxic T-lymphocytes (CTLs) after encountering a viral antigen for the first time [2-4]. The CTL responsiveness against a specific viral antigen is likely to vary between individuals, as well as within individuals over time (for example, at successive stages of HIV infection) [1]. The effectiveness of an anti-viral CD8+ response will depend on molecular factors such as the affinity of the T-cell receptor for the viral peptide in the context of Major Histocompatibility Complex (MHC) molecules, as well as MHC polymorphisms that determine which particular viral peptides are presented to the immune system [1,3,5].

At epidemiological (or population) levels, the importance of contact structure (or network connectivity) for disease transmission has long been acknowledged [6]. Locally structured networks can qualitatively alter infection dynamics through clustering behaviour with pairs of connected individuals sharing many common neighbours. The effects of population heterogeneity on infection spread are important but complex. Thus, when compared to well-mixed populations, local heterogeneous contact patterns can either slow or accelerate the progression of infection – depending on the structure of the network [6-14].

There are rich traditions of modelling centered specifically on the dynamics of infections at cellular [1,15] and population levels [2] that have profoundly advanced our understanding of disease dynamics and control. While the insights gained from these modelling techniques is remarkable, it is becoming evident that there are unique epidemiological processes of infectious diseases that are likely governed by the dynamics of the immune systems of individuals in a population (e.g., rebounds in the prevalence of some infectious diseases, antigenic variation and competition, waning immunity, and transient cross-immunity of sexually transmitted infections) [16]. Many of these may have significant consequences for creating optimum prevention strategies (e.g., vaccination or prophylactic chemotherapies) and establishing an adequate level of herd immunity.

In spite of the focused nature of current modelling applications, the need for integrating an immune system mechanism into epidemiological models has been recognized [17-19], and unified theoretical templates of these biological domains have been developed [20,21]. Although these initial immuno-epidemiological frameworks demonstrate innovation and clarity, they lack the representation of certain cellular components and immunological processes needed to characterize important epidemiological contexts such as antigenic variation, coinfection, and the immunological impact of prevention efforts. As a result, the link between host-pathogen interactions and their impact on the spread of infectious diseases across a population remains under-explored. Here, we present a simple mathematical framework that provides an alternate approach for unifying infection dynamics at the immune system and epidemiological scales. Although the analyses presented in this paper are almost entirely abstract, in the broadest context we advance the arguments that: one, individual immune response dynamics are important for shaping population-wide disease dynamics; and two, a modelling framework should not only be focused on a linked transmission system that can advance overall theoretical understanding, but also inform infection control decisions.

## Methods

### Combined model for infection dynamics

To gain insight into how the basic laws of viral dynamics, within an individual, will eventually affect the spread of a virus throughout a population of connected individuals, we considered a simple integrated model of the immune response and population structure. To this end, we elaborated on a simple, previously described model of the interactions between a replicating virus, host cells, and cells of the immune system specific for infected host cells (namely CD8+ T-lymphocytes) [1,4]. We have modified this framework by placing each individual in the population within a simple randomly-distributed (Poisson) network of 1000 people such that the viral load of a given individual is linked with the viral load of adjacent individuals within the network (described below). This basic model of anti-viral immune responses and population dynamics for each individual contains five variables: uninfected cells *x*_*i*_, infected cells *y*_*i*_, free virus particles *v*_*i*_, precursor CTLs (CTL_*P*_) (i.e., CD8+ cells that have recognized a specific antigen but lack specific effector functions) *w*_*i*_, and CTL_*P *_cells that differentiate and inhibit viral replication through cytotoxic effector activity (CTL_*E*_) *z*_*i*_.

Following Nowak and May [1] and Wodarz and colleagues [4], the emergence of uninfected cells occurs at a constant rate *λ*. Infected cells arise through contact between uninfected cells and free viral particles at a rate *βx*_*i*_*v*_*i *_and die at a rate *ay*_*i*_. A person's free virus load is produced by infected cells, at a rate *ky*_*i*_, and declines at a rate *uv*_*i*_. The rate of CTL_*P *_proliferation for each person in the population in response to antigen is given by *c*_*i*_*y*_*i*_*w*_*i*_. The parameter *c*_*i *_denotes the CTL_*P *_responsiveness, which is defined as the proliferation of specific precursor CTLs cells (i.e., CTL_*P *_cells) after their first encounter with a foreign antigen at the site of infection. While antigen is present, CTL_*P *_cells differentiate into CTL_*E *_cells at a rate *c*_*i*_*q*. In the absence of antigenic stimulation, each *i*th person's CTL_*P *_population decays at a rate *bw*_*i*_. Infected cells are killed by CTL_*E *_cells at a rate of *py*_*i*_*z*_*i*_. The parameter *p *specifies the rate at which CTL_*E *_cells kill infected cells. Once the infection is brought under control by the immune system, the CTL_*E *_population decays at a rate *hz*_*i*_.

To this model, we have added an additional term specifying that the rate at which a person's incoming flow of free viral particles is proportional to the viral load of their neighbours, *ω*_*i *_∑_*j*∈*P *_**A**_*ij*_*v*_*j*_. Here, *ω*_*i *_is the (typically very small) coefficient of connectedness that defines the weights on each of the connections between neighbours. We hereafter refer to *ω*_*i *_as the connectivity coefficient. The expression **A**_*ij *_is a randomly selected, symmetric, binary *n *× *n *adjacency matrix that describes "who is connected to whom". This matrix describes the structure of the Poisson-distributed network. The vector, *v*_*j*_, is the viral load of the *j*th network contact of person *i*, and *P *is the population. These assumptions lead to the following system of ordinary differential equations:

$${\dot{x}}_{i}$$ = *λ *- *x*_*i *_(*d *+ *βv*_*i*_)

$${\dot{y}}_{i}$$ = *βx*_*i*_*v*_*i *_- *y*_*i *_(*a *+ *pz*_*i*_)

${\dot{v}}_{i}$ = *ky*_*i *_+ *ω*_*i *_∑_*j*∈*P *_**A**_*ij*_*v*_*j *_- *uv*_*i*_

$${\dot{w}}_{i}$$ = *c*_*i*_*y*_*i*_*w*_*i *_(1 - *q*) - *bw*_*i*_

$${\dot{z}}_{i}$$ = *c*_*i*_*qy*_*i*_*w*_*i *_- *hz*_*i*_.

We numerically solved the above system of equations for each individual *i *in the population (*i *= 1, ..., 1000). The initial conditions that accompanied this system of equations for viral introduction were:

$$\begin{array}{c} \begin{array}{cc} x_{i}(0)=\lambda /d, & y_{i}(0)=\{\begin{array}{cc} 0.1, & \text{if }i=3\\ 0, & \text{otherwise} \end{array}, \end{array}\\ \begin{array}{cc} v_{i}(0)=\{\begin{array}{cc} 0.01, & \text{if }i=3\\ 0, & \text{otherwise} \end{array}, & \begin{array}{cc} w_{i}(0)=0.01, & \text{and }z_{i}(0)=0. \end{array} \end{array} \end{array}$$

In all simulation experiments, parameter values were based on those presented previously by Wodarz and colleagues [4] (see Table 1). Symbolic equilibrium analyses are presented in the Results section below.

**Table 1:** Parameter values that were used in the simulations of the basic model.

Parameter	Description	Value (units)	
*λ*	Production rate of uninfected cells	10.0	(cells/day)
*d*	Rate of uninfected cell die-off	0.1	(day-1)
*β*	Rate infected cells are produced from uninfected cells and free virus	0.01	(virion·day^-1^)
*a*	Infected cell death rate (due to virus)	0.5	(day^-1^)
*p*	Rate that infected cells are killed by CTL_*E *_cells	1.0	(cells/day)
*b*	Rate that CTL_*P *_die-off	0.001	(day^-1^)
*q*	Fraction of CTL_*P *_cells that proliferate into CTL_*E *_cells	0.1	(T-cell/T-cell)
*h*	Rate of CTL_*E *_die-off	0.1	(day^-1^)
*k*	Rate at which free virions are produced from infected cells	3.0	(virion·day^-1^)
*u*	Viral decay rate	3.0	(day^-1^)

Simulations were based on values used in Nowak and May [1], Nowak and Bangham [3] and Wodarz and colleagues [4]. Immune responsiveness (c_*i*_) and the connectivity coefficient (*ω*_*i*_) were varied throughout this paper. Their specific values for each simulation experiment are described in the Methods section.

For describing infection spread among the population, we used the mean and accumulated mean viral load as our main measure of infection prevalence. The accumulated mean viral load, *A*_*v *_(*t*), in the population was the integral of the mean viral load from the beginning of a given simulation (time 0) until time *t*, and was used as a proxy for the final size and severity of an outbreak. It was defined as $A_{v}(t)=\int_{0}^{t}\bar{v}(\tau )d\tau$, where $\bar{v}(t)=\frac{\sum_{i}v_{i}(t)}{\left|P\right|}$ is the mean viral load in the population at time *t*, and where |*P*| is the number of people in the population.

### Individual immune responsiveness

For experiments associated with parameter *c*_*i*_, we examined the effect of assuming specific values (homogeneous across the population) on infection spread. However, because individuals are likely to vary in their ability to respond to infection [4,5], we also conducted experiments in which the population was divided into two halves with different *c*_*i*_, and in which each individual's immune responsiveness was drawn from a truncated normal distribution with (*μ *= 0.063 and *σ*^2 ^= 0.0005) and confined to support over the interval [0.01,0.1]. Variance was estimated from the square of the interval divided by four: ${\left[\frac{0.1-0.01}{4}\right]}^{2}$. Our mean and range values were derived from the values studied by Wodarz and colleagues [4]. In all cases, values of *c*_*i *_were set at the beginning of the simulation, and remained static for the duration of that simulation.

### Weight of network connectivity between people and infection spread

One of the most obvious features of viruses is their capacity for person-to-person transmission [7]. Contact patterns provide important information for understanding the transmission properties of the pathogens, themselves, as well as where to concentrate prevention efforts [6]. Because exact values for the connectivity coefficient *ω*_*i *_will often vary over time [7], we assumed that *ω*_*i *_followed a random uniform distribution with mean, $\frac{\theta_{1}+\theta_{2}}{2}=0.5$ and variance, $\frac{{\left(\theta_{2}+\theta_{1}\right)}^{2}}{12}=0.083$. The value of *ω*_*i *_was dynamically varied for the majority of our analyses. Just as with immune responsiveness, the circumstances that focused on the specific effect of a person's connectivity, *ω*_*i *_was assigned a constant value for the entire population. High, moderate, and low values of *ω*_*i *_were arbitrarily assumed to be 1.0 × 10^-3^, 1.0 × 10^-6^, and 1.0 × 10^-9^, respectively.

### Time until re-infection and immunological memory

A direct consequence of an individual's ability to respond to and eliminate an infection is the formation of immunological memory. Within the host, memory CD8+ T-cell populations have the ability to rapidly elaborate effector functions to respond quickly and efficiently when re-exposed to infection. These properties of memory cells will not only decrease the duration of subsequent infection within the host, but their presence is considered to increase the level of herd immunity in a population [22,23]. And yet, the generation of memory T-cells exhibits both antigen-dependent and antigen-independent characteristics [4,24]. This appears to rely on the time scale of the infection being studied: antigen-independent immunological memory has largely been observed in acute infections, while antigen-dependence has been observed in the context of persistent infections [25].

To examine the effect of re-infection on the accumulated viral load in the population, we considered two different scenarios. Scenario one was after an acute infection that was completely cleared by the immune system and where memory CTLs (here a proportion of CTL_*P *_cells) persist for long periods of time in an antigen-independent environment. Scenario two was for a low-grade persistent infection characterized by a high acute-phase viral load followed by a reduction to very low levels but not complete elimination. Specifically, this involved re-introducing infection at a disease-free equilibrium (see below), where viral antigen has been eliminated (scenario one), and comparing it to re-introducing infection near an endemic equilibrium (see below), where viral antigen has persisted at low levels (scenario two). For all re-infection experiments, both the population and an individual were separately re-infected at time *t *= 9000 days with a viral load that is equal to the initial amount of virus, *v*_*i *_(0). We also investigated periodically re-infecting the population and an individual at *t *= 1000, 3000, 6000, and 9000 days. For each scenario, the values of *c*_*i *_(immune responsiveness) and *b *(rate of CTL_*P *_die off) assumed values according to Wodarz and colleagues [4] for the comparison of antigenic persistence and elimination. Here, individuals were assumed to be strong responders *c*_*i *_= 0.1, and have a slow rate of CTL_*P *_die off *b *= 0.001.

Because our basic model is deterministic and was originally used to describe persistent viral infections [3], CTL_*E *_responses cannot reduce both *v*_*i *_(*t*) and *A*_*v *_(*t*) → 0. Therefore, following Wodarz and colleagues [4], for scenario one (above) we defined a threshold value where virus, although likely at low levels, was considered extinct, *v*_*ext*_. For our simulations of long-term dynamics that assumed that the virus was eliminated, our extinction threshold was chosen (arbitrarily) to be marginally larger than the endemic equilibrium value ${\hat{v}}_{i}$ = 0.013. Here *v*_*ext *_= 0.015.

### Varying the infecting dose

The outcome of viral infection, in general, is thought to be related to the size of the infecting dose a person initially receives [23]. Therefore, we also investigated the impact of varying the infecting doses a person received from their network contacts. More specifically, we examined the situation of ${\dot{v}}_{i}$ = *ky*_*i *_+ *ω*_*i*_*φ *∑_*j*∈*P *_**A**_*ij *_*v*_*j *_- *uv*_*i*_, where *φ *is the constant for the infecting dose received by a person from their network contacts, with *φ *= 1 being the default value. These experiments allowed to us to obtain an initial understanding of the dynamical behaviour of the model under different viral quantities transmitted throughout the population. For these experiments a person's immune responsiveness, *c*_*i*_, was a static random variable and the network connectivity coefficient, *ω*_*i*_, was a stochastically-varied random variable.

## Results

### Equilibrium analyses

For a single-person where **A**_1,1 _= 0, the equations in the basic model are associated with three equilibria. The first is a disease-free equilibrium in which free virus, infected cells, CTL_*P*_, and CTL_*E *_cells are all absent, and only uninfected cells are present: $\hat{x}=x(0)=\frac{\lambda }{d},\hat{y}=\hat{v}=\hat{w}=\hat{z}=0$. This equilibrium is unstable for the scenario in which viral antigen persists, but is locally stable when viral antigen is eliminated. The second equilibrium is a stable endemic equilibrium, in which free viral particles and infected cells are in balance with uninfected, CTL_*P*_, and CTL_*E *_cells:

$$\begin{array}{l} \hat{x}=\frac{\lambda uc(1-q)}{duc(1-q)-\beta kb}\hat{y}=\frac{b}{c(1-q)}\hat{v}=\frac{kb}{uc(1-q)}\\ \hat{w}=\frac{h\left({\left(1-q\right)}^{2}(\beta \lambda ck-aduc)-a\beta kb(q-1)\right)}{qbp(duc(q-1)+\beta kb)}\text{ and }\hat{z}=-\frac{(aduc-\beta \lambda ck)(q-1)-a\beta kb}{pduc(q-1)-p\beta kb}. \end{array}$$

The final equilibrium is an unstable "defense-free" equilibrium in which free viral particles, uninfected cells, and infected cells are present, but at which CTL_*P *_and CTL_*E *_cells are absent:

$$\hat{x}=\frac{ua}{\beta k},\hat{y}=\frac{uad-\lambda \beta k}{a\beta k},\hat{v}=\frac{uad-\lambda \beta k}{ua\beta },\text{ and }\hat{w}=\hat{z}=0.$$

The equilibria described above for a single-person have a close relationship with the equilibria for a connected multi-person population. For a multi-person population, the number of equilibria for our basic model rises geometrically with population size. While the count and stability of these equilibria differ significantly for the cases of antigenic persistence and elimination, two equilibria are shared by both scenarios: the first is a unique disease-free equilibrium, in which the values of the state variables for each individual in the population are identical to those under the single-person disease-free equilibrium.

Compared to the corresponding single-person equilibrium, this multi-person equilibrium is unstable for the case in which viral antigen is assumed to persist, but is locally stable for the case in which a viral antigen is eliminated; the second is a unique stable endemic equilibrium, in which the values of the state variables for each individual in the population are very close to those that would obtain for a single-person endemic equilibrium, but are slightly offset due to the small rate of virions transmitted by neighbours. For example, given a very high coupling coefficient (*ω*_*i *_= 0.001), the difference of viral levels between the single-person and multi-person endemic equilibrium is only 3 per cent for an individual with 5 neighbours (not shown). The exact formula for each equilibria value, of each individual, will depend on population size and network structure; because of this dependence, and because the equilibria for each individual within a multi-person population are similar to the corresponding single-person equilibrium, we do not describe a general formula here.

The number and stability of the remaining equilibria beyond the two just described depend on whether viral antigen is assumed to be eliminated. If antigen persists, and we ignore all non-physical equilibria associated with negative values of state variables, a total of 2^|*P*| ^+ 1 distinct equilibria will be associated with a population of size |*P*|. In addition, there is a set of unstable 2^|*P*| ^- 1 "combinatorial" equilibria in which some individuals are in a state very close to the defense-free equilibrium or to the endemic equilibrium for the single person case. Thus, each such population-wide unstable equilibrium is essentially a simple superposition of the single-person defense-free and endemic equilibria. As in the single-person case, the endemic equilibrium is the sole stable equilibrium.

For a model that assumes viral antigen is eliminated, the structure and stability of the equilibria are significantly different. Recall that for a given non-zero virus extinction threshold, the disease free equilibria for each individual in isolation and for the population as a whole are locally stable. However, if a virus is driven extinct within a person, any finite-rate perturbations to the viral load in that individual disease free equilibrium will be insufficient to elevate their viral load, and will therefore maintain complete extinction of the virus. A given individual who has undergone viral clearance will therefore remain virus-free even in response to coupling with neighbours. As a result, a population of size |*P*| will exhibit 3^|*P*| ^equilibria. Specifically, for different individuals this will include both 2^|*P*| ^globally stable endemic and disease-free equilibria and 3^|*P*| ^- 2^|*P*| ^unstable defense-free equilibria.

### Simulation experiments

#### Immune responsiveness limits viral transmission

**Figure 1:**
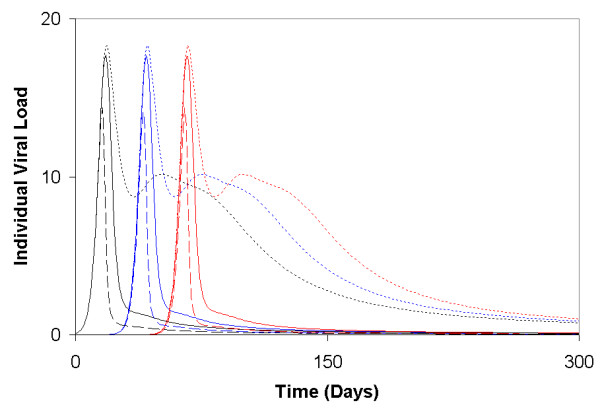
**Evolution of individual viral load of infected cases and their network contacts**. For illustrative purposes, results displayed here are for three people in the population. Person 3 (black lines) and Person 1 (blue lines) are connected, and Person 1 and Person 2 (red lines) are connected. Here, *c*_*i *_= 0.01 (dotted lines), 0.05 (solid lines), and 0.1 (dashed lines) (Here *v*_*ext *_= 0.015 and *ω*_*i *_was assumed to be a uniformly distributed random variable).

**Figure 2:**
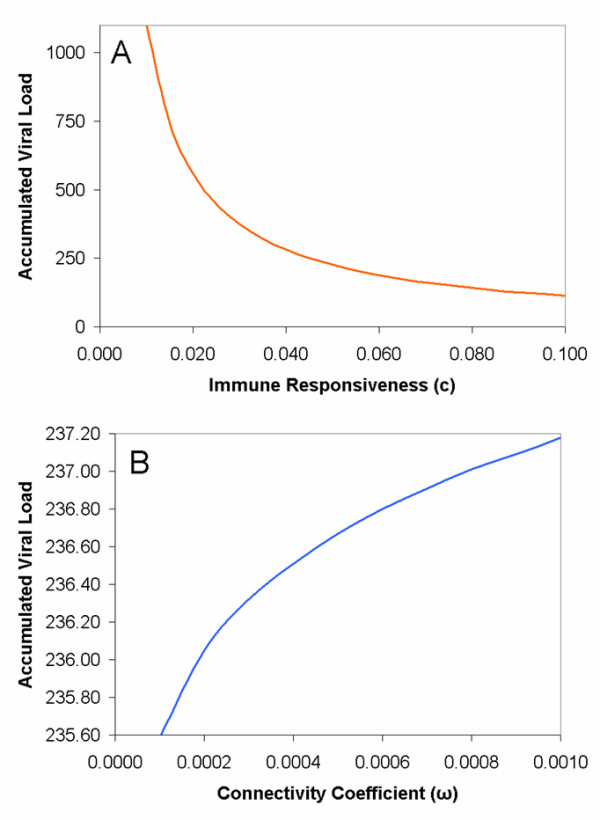
**Variations in parameter values and their effect on the population-wide accumulated viral load**. Additional parameter values investigated when studying the effect of (A) immune responsiveness and the connectivity coefficient (B) on the population-wide accumulated viral load.

**Figure 3:**
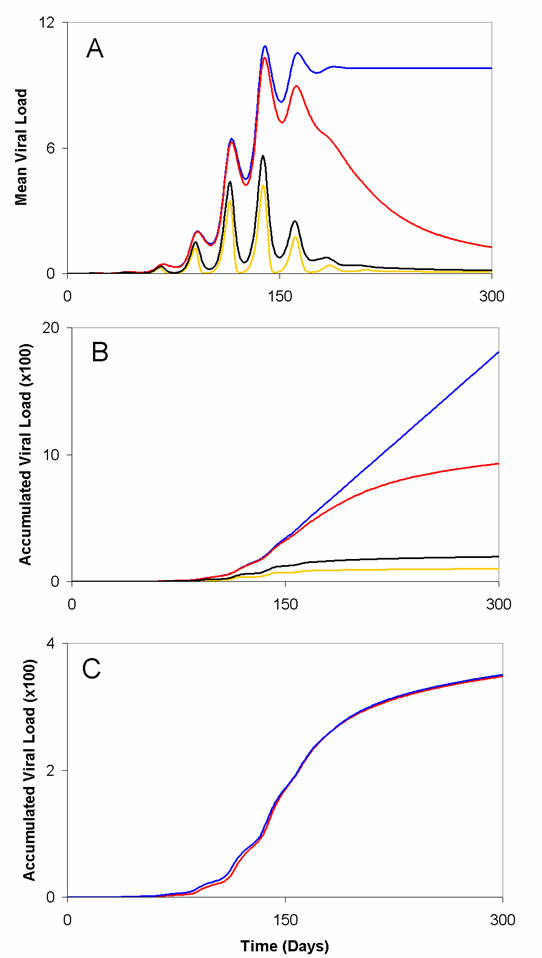
**The impact of a person's immune responsiveness for the short-term dynamics of an outbreak**. (A and B) A comparison between the immune responsiveness and the overall behaviour of an outbreak (A), as well as the overall severity an outbreak (B), as measured by the mean and accumulated viral load in the population, respectively. Mean and accumulated viral loads were computed from simulating our basic model for constant values of immune responsiveness: *c*_*i *_= 0.001 (blue line), 0.01 (red line), 0.1 (yellow line), and random uniformly distributed (black line). (C) Assuming that the population is composed of an equal proportion of stronger *c*_*i *_= 0.1 and weaker responders *c*_*i *_= 0.016, the model was simulated to study the effect on the accumulated viral load in the population by starting the infection in the sub-population of stronger responders (red line) and weaker responders (blue line). These experiments demonstrate no clear correlation between viral load and starting an infection in either strong or weak responders. For scenarios (A, B, and C) the connectivity coefficient, *ω*_*i*_, was a stochastic random variable. All other parameter values were based on values presented by Wodarz and colleagues [4] and are displayed in Table 1.

The abundance of virus – that is, the viral load – is an important correlate of pathogenicity and disease progression of many viral infections [3]. Our integrated model both reproduced the well-known relationships between an individual's immune responsiveness *c*_*i *_and their viral load (Figs. 1 and 2) [1,4], and demonstrated the implications of this relationship to the short-term dynamics of an outbreak (Fig. 3). Overall, a population that possesses a high value for *c*_*i *_will reduce the scale and overall severity of an outbreak when compared to a population of weaker responders (Fig. 3A and 3B). Interestingly, these results demonstrate a correlation between immune responsiveness and the natural history of infection in the population. For populations of strong responders, infection is eliminated (or at least depleted to very low levels), whereas in a population of weak responders infection is likely to become endemic (Fig. 3A). If we assume that a population is composed of a combination of strong and weak responders, then starting an infection in either a weak (low *c*_*i*_) or strong (high *c*_*i*_) responder, interestingly, had no significant impact on the overall severity of an outbreak (Fig. 3C). More realistic assumptions of heterogeneity, in which a person's immune responsiveness is drawn from a random normal distribution, resulted in a lower viral load in the population. On the whole, these experiments suggest that increasing the disparity between people's ability to respond to an infection, while maintaining an average rate may worsen the overall impact of an outbreak within that population (Fig. 3A and 3B).

#### Network connectivity affects the time between peaks in the viral load

**Figure 4:**
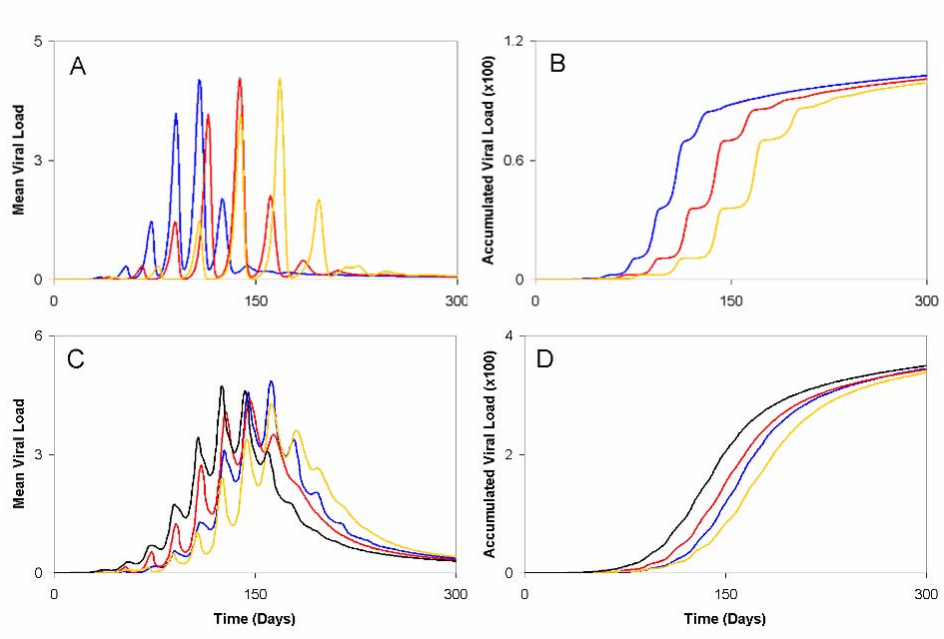
**The transmission of virus across the population differs for variations in the connectivity coefficient, *ω*_*i*_**. (A) Higher values of the connectivity coefficient (*ω*_*i *_= 1.0 × 10^-3^) shortened the time required to spread the disease through the population, as well as the peak of the outbreak (blue line). Lower values of the connectivity coefficient (*ω*_*i *_= 1.0 × 10^-4 ^and 1.0 ×10^-5^) had the opposite effect (red and yellow lines, respectively). (B) Both high and low values of *ω*_*i *_demonstrated no apparent sizeable relationship with the accumulated viral load in the population (colour code the same as 3A). For scenarios (A and B) a person's immune responsiveness was randomly determined from a random normal distribution with *μ *= 0.063 and *σ *= 0.0225 (see Methods for further details). For scenarios (C and D), immune responsiveness for fixed values of *c*_*i *_= 0.1 and 0.016 were combined in simulations with different fixed values of *ω*_*i *_= 1.0 × 10^-3 ^and 1.0 × 10^-5^. The colour code is the same for 3A.

**Figure 5:**
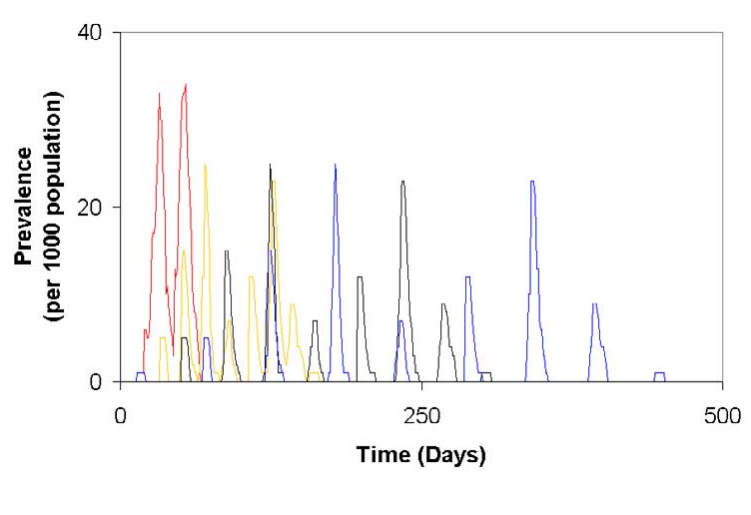
**Prevalence of a disease (per 1000 population) based on different values of *ω*_*i*_**. Here, *ω*_*i *_= 1.0 × 10^-1 ^(red curve), 1.0 × 10^-3 ^(yellow curves), 1.0 × 10^-6 ^(black curves), and 1.0 × 10^-9 ^(blue curves).

Varying the magnitude of peoples' connectivity coefficient *ω*_*i *_in our model re-produced previously described behaviour of infection spread, and therefore built confidence in our model structure with respect to previous discussions of contact patterns [6-8,14] (Fig. 4). High values for *ω*_*i *_reduced the time until the peak of an outbreak as well as the timing between peak viral levels in neighbouring individuals, while infection spread was delayed among the population when values of *ω*_*i *_were low (Fig. 4A). Given these particular assumptions regarding the strength of connectivity among individuals, it is also likely that delays in disease progression (demonstrated by an increased period between oscillatory peaks) will be observed. With larger values of *ω*_*i*_, the numbers of peaks and troughs in the prevalence are reduced, and begin to merge into a more continuous (and more familiar) outbreak pattern (Fig. 5). While changing *ω*_*i *_changes the rate with which the population-wide viral load accumulates, it has little impact on the asymptotic limit of that viral load (Fig. 4B).

**Figure 6:**
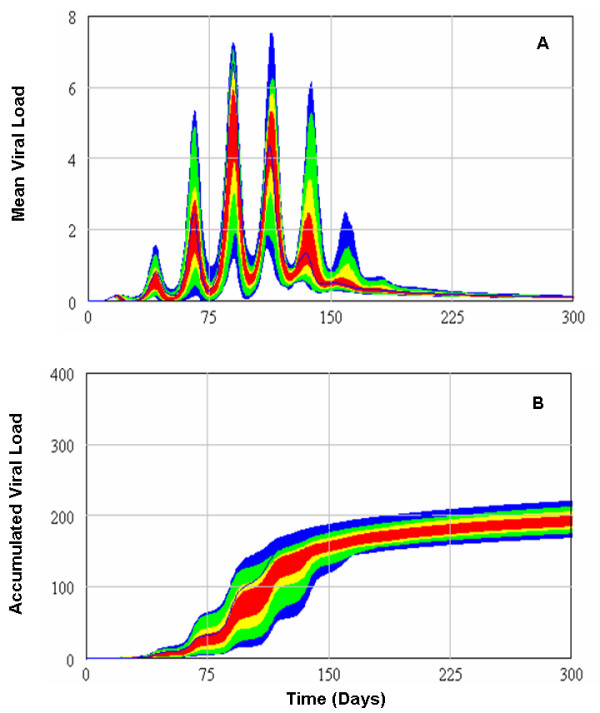
**Mean (A) and accumulated (B) viral loads in the population after 100 Monte Carlo realizations**. Each realization is associated with a randomly selected Poisson network, as well as a randomly selected value of immune responsiveness (drawn from a normal distribution) and distinct stochastic trajectories for network connectivity coefficients (drawn from a uniform distribution).

Our present methodology also allowed us to investigate, in the context of different combinations of immune responsiveness, the impact of a person's connectivity coefficient *ω*_*i*_, on infection spread in a population. These considerations demonstrate, rather intuitively, that the peak mean viral load and the subsequent accumulated viral load in the population will decrease for a combination of low connectivity and high immune responsiveness, while increasing for high connectivity and low immune responsiveness (Fig. 4C and 4D). Furthermore, performing 100 Monte Carlo iterations across randomly varied parameter values for immune responsiveness, the connectivity coefficient, and randomly generated network structures highlighted that the above results are likely to be quite robust for many different combinations of parameter values (Fig. 6).

#### Re-infection, immunological memory, and herd immunity

**Figure 7:**
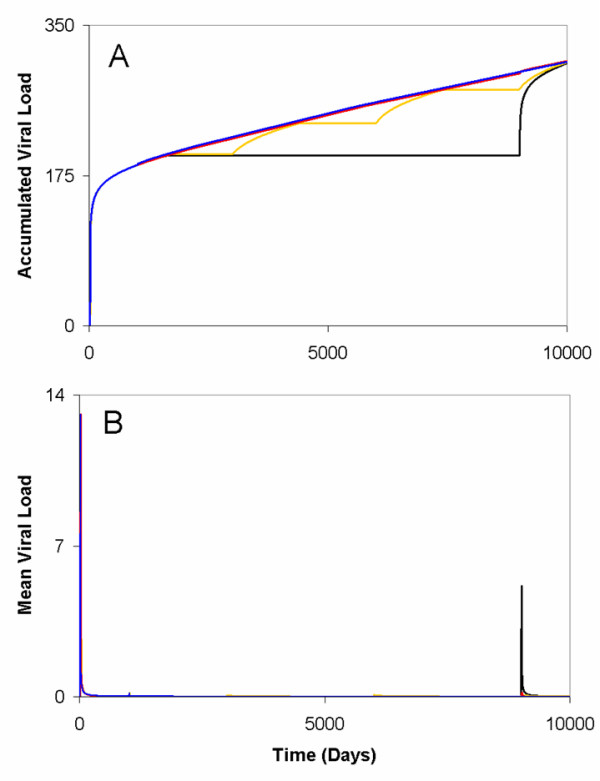
**Viral dynamics for re-infection to antigen when it is eliminated compared to when it persists**. Antigen was re-introduced to the whole population, at *t *= 1000, 3000, 6000, and 9000 days (yellow and blue lines), or at a single time step (*t *= 9000 days) (black and red lines) under the assumption of antigenic elimination and antigenic persistence, respectively. Here, *ω*_*i *_= 0.1, and a *v*_*ext *_= 0.015 was used in antigenic elimination simulations. (A) With the exception of antigenic persistence (red and blue lines), re-infection for the population at different intervals produces qualitatively different behaviour than antigenic elimination (yellow and black lines). However, the asymptotic accumulated viral load in the population is similar, regardless of whether or not antigen persists or is eliminated. (B) These qualitative differences are also observable for the mean viral load in the population. Assuming either scenario one or two, a small positive growth in the mean viral load following re-infection at *t *= 1000, 3000, 6000 days (yellow line), and at *t *= 9000 days (black and red lines) occurs.

**Figure 8:**
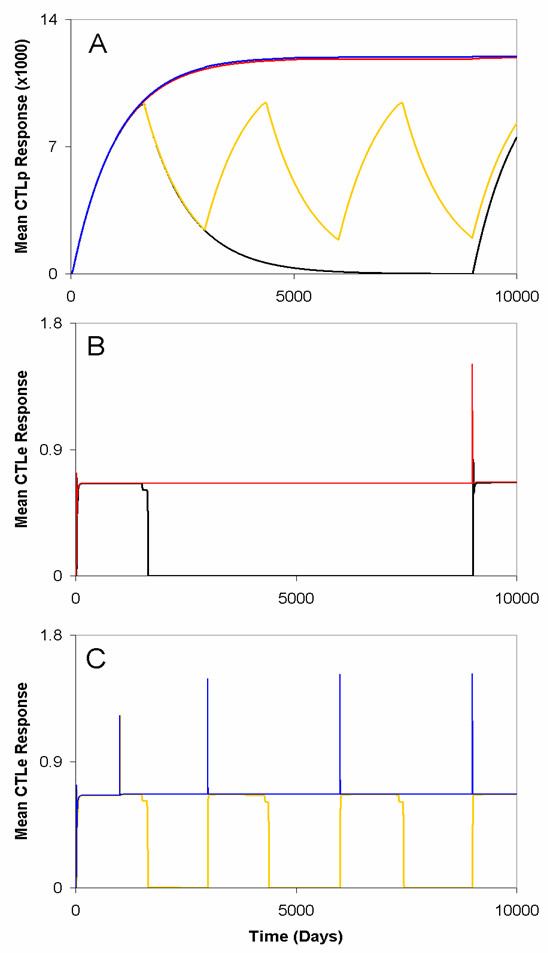
**Immune system dynamics for re-infection when viral antigen is eliminated compared to when it persists**. Here, the same re-introduction protocol as for Fig. 5 was followed. (A) Antigenic persistence (red and blue lines) keeps CTL_*P *_abundance continually high regardless of when antigen is re-introduced repeatedly at *t *= 1000, 3000, 6000, and 9000 days (blue line) or only once at *t *= 9000 days (red line). Antigenic elimination (with slow rates of CTL_*P *_decline, *b *= 0.001 day^-1^, high immune responsiveness, *c*_*i *_= 0.1, and assumed *v*_*ext *_= 0.015) demonstrates that re-expansion requires time for individuals to mount an effective immune response (yellow and black lines). (B and C) There is also a proportional, positive growth in the abundance of CTL_*E *_cells that follows directly from the expansion of CTL_*P *_cells after single instance of re-introducing viral antigen (B) assuming antigen is eliminated (black line) or antigen persists (red line), as well as repeated re-introduction (C) assuming antigen persistence (blue line) and antigenic elimination (yellow line).

Figures 7 and 8 present the simulation experiments for re-infection. Under scenario one, our model indicates that the longer the period until re-infection, the larger the post-exposure mean viral load in the population will be (Fig. 7A). This reflects that, as the time prior to re-infection increases, the CTL_*P *_populations are likely to decline towards naive levels and approach the disease-free equilibrium. With increasing time until re-infection, an individual will require a longer time to mount an effective immune response to reduce the severity of that re-infection (Fig. 8A). For scenario two (i.e., viral antigen persists after primary exposure), the recovered population does not experience positive viral growth if the virus is reintroduced (Fig. 7B). Therefore, any re-infection that is likely to occur will result in immediate inhibition of viral particles, and no considerable infection will take hold. What is interesting is that the asymptotic accumulated viral load from re-infection is essentially the same regardless of antigenic requirements or whether re-infection occurs repeatedly over time or infrequently later in time (Fig. 7B).

**Figure 9:**
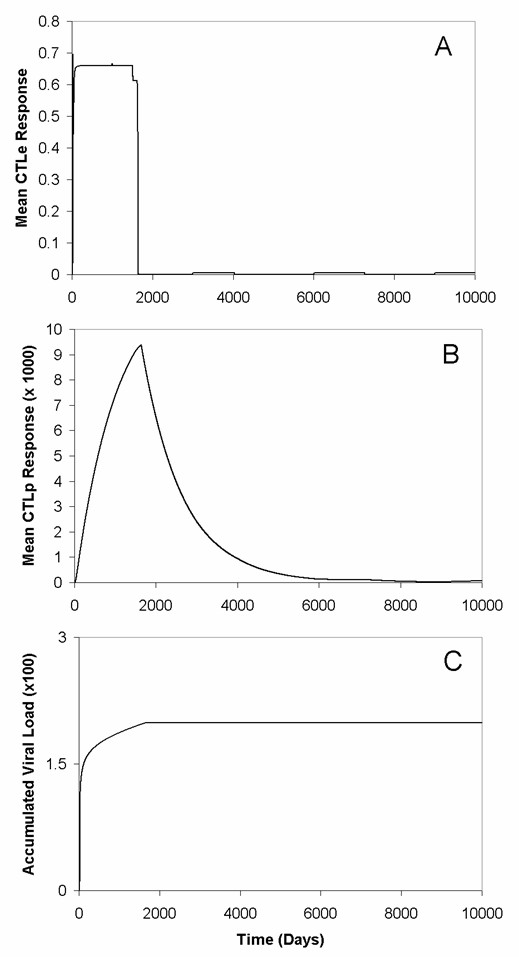
**Having people's immune systems primed through re-infection prevents infection from reaching the rest of the population**. Having key core people's immune system primed against re-infection (A and B) causes them to serve as barriers that prevent an outbreak from reaching the rest of the population, as measured by the accumulated viral load (C).

Notably, having key core people's immune system primed against re-infection causes them to serve as barriers that prevent that infection from reaching the rest of the population (Fig. 9A). We expect this to be because by time *t *= 9000 days, one person possess an elevated level of virus-specific CTL_*P *_cells (Fig. 9B) and will be able to easily increase the abundance of CTL_*E *_cells (Fig. 9C). Thus, this person is able to (almost instantaneously) clear the infection when it is reintroduced at *t *= 9000 days. This interestingly implies that, given the assumptions used in the model here, re-infecting key core people can be beneficial to the population.

### Variations in the infecting dose

**Figure 10:**
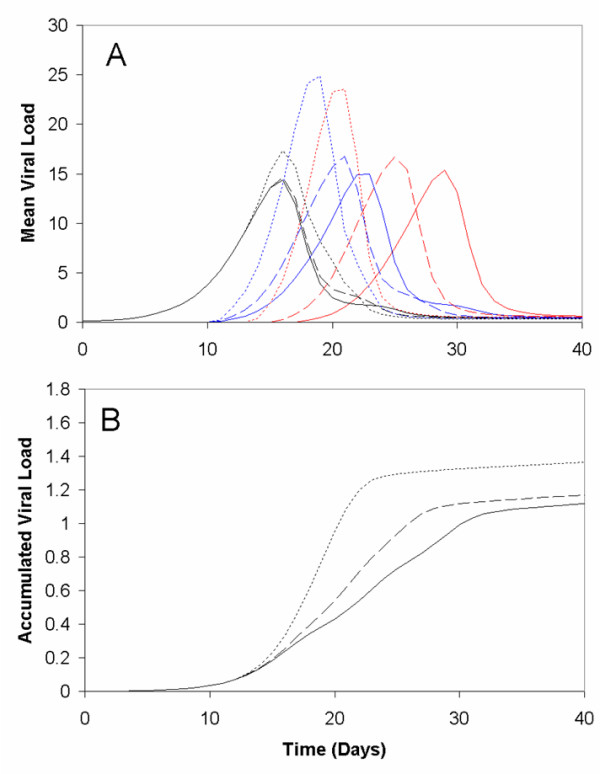
**Simulations of increasing the viral load transmitted to a person from their network contacts**. Individual viral loads (A), and accumulated viral load in the population (B) for a two- (dashed curves) and five-fold (dotted curves) increases in the quantity of free viral particles transmitted from a person's neighbour, compared to the simulations of the basic model used in the main text (solid curves). Again for illustrative purposes, the results in (A) are displayed for the same three individuals used in Fig. 1: Person 1 (blue curves), Person 2 (red curves), and Person 3 (black curves).

As expected, increases to the constant *φ *resulted in an increase in a person's viral load. It bears noting that, increasing the viral load incoming from a person's neighbour also appeared to have a similar effect on the timing of a person's peak viral load (i.e., larger values of *φ *lead to tighter spacing in time between the peaks in viral load of connected individuals) (Fig. 10A). However, this change in behaviour at the individual level did not appear to have quite the same impact at the population level, as there was no substantial change in the asymptotic behaviour of the accumulated viral load (Fig. 10B).

## Discussion

Future infectious disease research would benefit by striving to not only understand the properties of the invading microbe, or the body's response to infections [5], but also how individual responses affect the propagation of an infection throughout a population. Whilst this is not the first attempt to explicitly combine the nonlinear dynamics of immune reactions within individuals and the overall nonlinear dynamics of the interaction between an infection and a population of hosts, previous frameworks are better adapted to understanding very specific aspects of viral infections, such as re-exposure to viral antigen [20] and the role of memory T-cells in clearing reinfection [21]. In our opinion, our framework complements such previous contributions by incorporating a more detailed representation of the mechanisms of antiviral immune response, and thus will contribute towards improved understanding the immuno-epidemiological dynamics of viruses and other intracellular pathogens.

These initial results reinforce how coupling principles of immunology with epidemiological mixing provide a multi-scaled description of the relational aspects of an ecological system. In the short-term, the immune responsiveness of the population as a whole produces some very well-defined emergent properties and thus is likely to determine the natural history of disease in that population [21]. That is, there exist levels of immune responsiveness whereby a population of connected individuals will be able to eliminate a viral infection, while at others, it will likely become endemic. Interestingly, these emergent properties of our model demonstrate consistency with both traditional susceptible-infectious-removed properties (for populations with higher values of immune responsiveness) and susceptible-infectious-susceptible properties (for populations of weaker responders) within the clusters of people in the population even though these compartments were not explicitly defined (see Figs. 3A and 5). They also reproduce well-known dynamics of re-infection in a population after long periods of time [2], as well as intuition-based observations of how host-pathogen interactions influence herd immunity [22,24]. However, because these population-based results stem from an explicit description of the immune system, hypotheses relating the production of immunological memory to the long-term effects of re-exposure on the population can now be mathematically formulated and studied.

Another interesting result from this particular system is that the asymptotic accumulated viral load after re-infection is essentially conserved regardless of whether the virus is eliminated, if it persists, or whether re-infection occurs repeatedly over time or infrequently later in time. This conservation property reflects the fact that given the same starting point in state space, the value of *z *(*t*) and *w *(*t*) depends only on the integral of the count of infected cells *y *from 0 to *t*, and not on the specific trajectory taken by *y *within that interval. Conservation of morbidity within the population also raises a potentially important (and possibly controversial) question when it comes to creating control strategies, particularly for recurrent diseases such as influenza: is preventing population-wide reinfection until later in time that much more effective than having continual population-wide reinfection over time when the end results are likely to be similar?

Our methodology has made several simplifying assumptions that should be investigated. We imposed neither viral load thresholds required for contagion, nor any difference or quantization in the infecting dose people received. Although the outcome of viral infection, in general, is thought to be related to the size of the infecting dose a person initially receives [18], we found that our results were robust against variations in this parameter (see Fig. 10). Investigating the impact of different network structures (e.g., scale-free and small-world networks) is an important area of ongoing work.

Following Nowak and May [1], we have also assumed a basic model for virus dynamics. Because of the known role of CD8+ T-cells in the elimination of virally-infected host cells (e.g., influenza A infections [26-29], or adenovirus infections [30]), we have focused our discussion of immune responsiveness on CTLs, and thus ignored other types of innate and specific immunity. Our focus on CTL-mediated viral elimination was, largely, an attempt to establish plausibility of the multi-scale methods presented, not necessarily their complete adherence to immunological reality; the cytotoxic properties of activated CD8+ cells for clearing a viral infection are certainly not the whole story, and other immune responses are likely to affect the production of free virus. It should be noted, however, that the effect of other immune responses can be described in terms of this basic model by modifying its existing parameters. For example, production of cytokines by CD4+ T_*H *_cells are likely to reduce the infectivity parameter *β *and/or the rate at which infected cells are produced, *k*, while the role of neutralizing antibody- or complement-mediated responses may also enhance the removal rate of free viral particles, *u *[1]. Although considering other immune responses is assumed to have an additional influence on the viral dynamics at population levels [31,32], previous research at the individual level suggest that they are associated with qualitatively similar dynamics to those governing CTLs [1,3,4]. However, explicitly describing the cooperative interactions between CTLs and other immune responses, in the form of additional state equations, and their effect on the transmission of specific microparasite infections is also an important area of ongoing study.

## Conclusion

Despite the extensive use of mathematics in epidemiology, many theoretical challenges remain [33]. To improve our understanding of infectious diseases, future research will require theoretical tools that incorporate immunological and epidemiological features into a unified template [16,18]. Our goal in this paper was to expand upon the utility of merging aspects of immunology and epidemiology into a single conceptual framework. This analysis has produced some interesting and potentially important conclusions. We anticipate this framework to be a step towards articulating an overall, integrated, and more refined epidemiological theory that simultaneously describes broad categories of diseases dynamics at both cellular and organismal levels. Under a unified framework, continued molecular research on disease pathogenesis and host-pathogen interactions will likely have a reciprocal influence on epidemiological theory. Ideally, improvements to these combined theoretical templates will prove useful for the prediction of future trends in infectious disease epidemiology. Such combined methodologies could also lead to novel insights into understanding microparasite evolution and its role in disease virulence and persistence. Ultimately, these initial findings suggest that there are important immunological consequences to consider when designing effective interventions to control new variations of familiar diseases.

## Competing interests

The author(s) declare that they have no competing interests.

## Authors' contributions

D.M.V. and N.D.O. contributed equally to the writing of this manuscript and both have approved the final version.
